# Cap Polyposis: A Case Report

**DOI:** 10.31729/jnma.8177

**Published:** 2023-06-30

**Authors:** Tunam Khadka, Ganesh Kumar Giri, Pasang Sherpa, Nischal Shrestha, Sanjaya Poudyal

**Affiliations:** 1 Kathmandu Medical College and Teaching Hospital, Sinamangal, Kathmandu, Nepal; 2 Department of Internal Medicine, Kathmandu Medical College and Teaching Hospital, Sinamangal, Kathmandu, Nepal; 3 Department of Internal Medicine, Nepal Medical College and Teaching Hospital, Jorpati, Kathmandu, Nepal; 4 Department of Surgical Gastroenterology, College of Medical Science and Teaching Hospital, Bharatpur, Chitwan, Nepal; 5 Department of Surgery, Patan Academy of Health Sciences, Lagankhel, Lalitpur, Nepal

**Keywords:** *case reports*, *granulation tissue*, *inflammatory bowel diseases*, *polyps*

## Abstract

Cap polyposis is a gastrointestinal disease with multiple inflammatory polyps between the distal colon and rectum. Its symptoms overlap with inflammatory bowel disease with typical endoscopic features of multiple sessile polyps in the rectum and sigmoid colon, located at the apices of transverse folds. Microscopically, the polyps consist of elongated, tortuous, and distended crypts covered by a "cap" of inflammatory granulation tissue. In this report, we present a case of a 18-year-old male patient who underwent polypectomy for polyposis in multiple settings. He presented with one year of painless rectal bleeding and polyposis in a recto-sigmoid area on colonoscopy, with a single polyp in the sigmoid area and multiple polyps in the rectum. He was managed with immediate and interval polypectomy. Though cap polyposis is rare, it can be cured as it is laparoscopically resectable.

## INTRODUCTION

Cap polyposis is a rare condition characterized by erythematous, inflammatory colonic polyps covered by a cap of fibrinopurulent mucous.^1^ They are usually multiple but can be solitary and confined to the distal colon.^2^ Since the disease entity is rare and very unfamiliar to the physician, the exact aetiology and its pathogenesis have not been clearly defined yet. However, abnormal colonic motility leading to mucosal prolapse, infectious pathology associated with *Helicobacter pylori,* a history of pelvic surgery, and the altered intestinal microbiome are some evidence to explain its clinical origin. The most common symptoms are abdominal pain, tenesmus, bloody diarrhoea with mucus, and rectal bleeding.^3^ Here, we present an interesting case of 18 years male patient with cap polyposis.

## CASE REPORT

**Figure 1:**
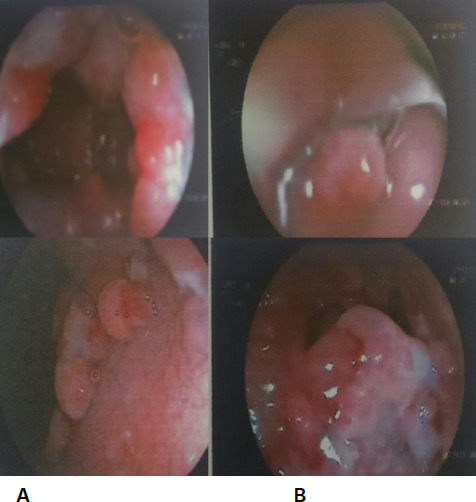
Colonoscopy with A) multiple rectal polyps and B) single polyps in the sigmoid colon.

A 18-year-old male was referred to our tertiary health care centre for evaluation of painless per rectal bleeding for one year. Our patient had been in his usual state of health before the current presentation of painless rectal bleeding, however, there was no history of altered bowel habits, per rectal mass, mucus deposit on the stool, abdominal pain, weight loss, anorexia, vomiting, chest pain, fever, or burning micturition. His past medical history and family history were insignificant. He had no significant comorbidities like diabetes mellitus, hypertension, and tuberculosis. His systemic examination at presentation was normal including per abdominal examination. However, a digital rectal examination revealed multiple rectal polypoidal growths 4 cm from the anal verge, and the gloved finger was not stained with blood. Proctoscopy did not show the presence of haemorrhoids or ulcers. His initial workup on presentation which included blood and urine investigations was within normal limits with a CEA of 1.24 ng/ml. However, stool routine examination and stool showed occult blood. He underwent a colonoscopic biopsy that revealed multiple rectal pedunculated polyps scattered all over the lower rectum with a normal upper rectum and a single sigmoid polyp (Figure 1).

**Figure 2:**
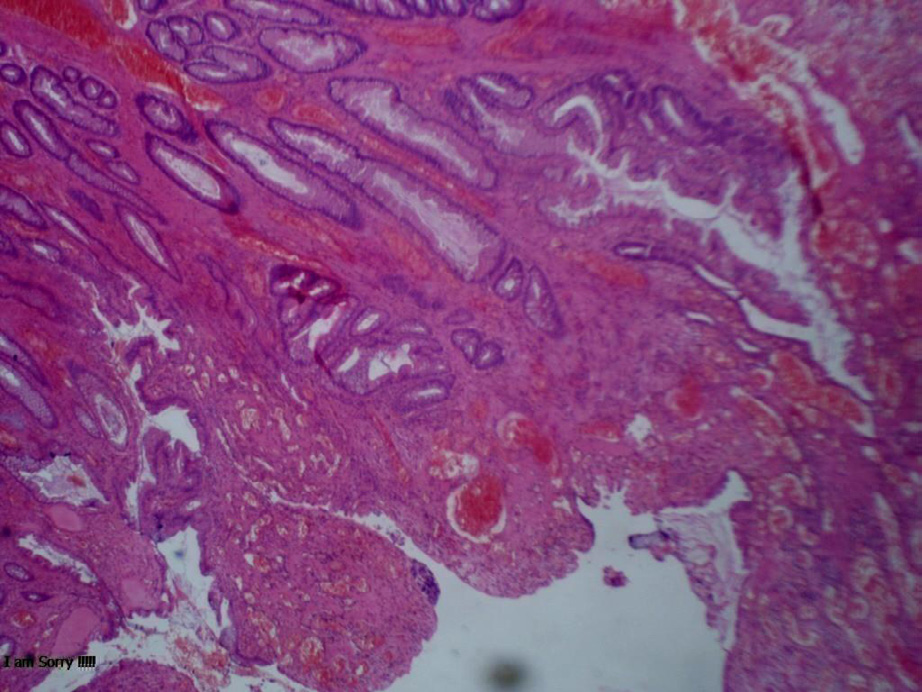
HPE showing CAP polyposis.

Upper gastrointestinal endoscopy was normal. Histopathologic examinations (HPE) revealed polypoidal mucosa with surface ulceration and granulation tissue forming an inflammatory cap and mixed inflammatory infiltrate suggestive of the cap polyposis (Figure 2).

Contrast-enhanced computed tomography (CECT) of the abdomen and pelvis showed heterogenous enhancing soft tissue mass in the rectum about 3.5 cm above the anal verge with normal peri-rectal fat, mesorectal fascia, and no regional and distal abdominal lymphadenopathy.

**Figure 3:**
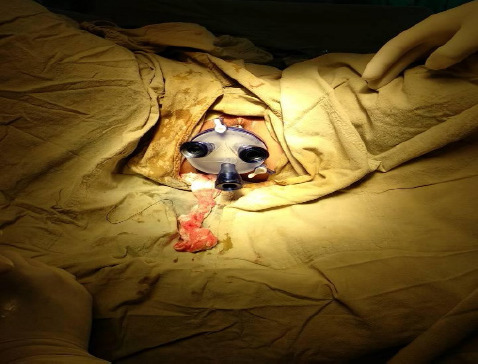
Specimen retrieved using TAMIS.

After educating the patient on risks, benefits, and management alternatives, consent was taken, and we planned for the polypectomy under anaesthesia using the transanal minimally invasive surgery (TAMIS) platform. On the day of admission, the patient's vitals were normal with elevated WBC counts of 11400/mm^3^. Other blood parameters were within normal limits. Polypectomy and submucosal dissection were done up to 10 cm using the GelPOINT Path Transanal Access Platform from the anal verge with the approximation of mucosa and the raw area (Figure 3).

Numerous pedunculated and sessile polyps of varying sizes just above the dentate line were excised. The excised polyp was sent for a histopathology examination. Then, our patient was discharged with advice to follow up in two weeks, on the first postoperative day with stool softeners and seven days course of oral ciprofloxacin 500 mg twice a day and oral metronidazole 400 mg three times a day. On follow-up after two weeks, sigmoidoscopy was done which showed polyposis starting just above the dentate line in the anal canal with a significant reduction in polyp number and size than the previous one and scattered sessile polyps in the rectum.

**Figure 4:**
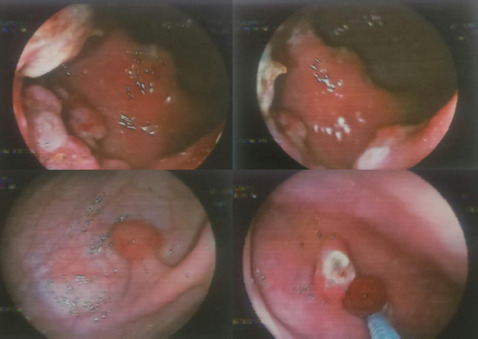
Snare polypectomy of the sigmoid colon.

A single pedunculated polyp was removed with a snare polypectomy and discharged with a plan for a second session of excision after four months (Figure 4).

After four months, he was arranged for second session excision by TAMIS. He was discharged on the first postoperative day with stool softener and oral metronidazole 400 mg three times a day for 5 days, and planned for review sigmoidoscopy after three months.

## DISCUSSION

Cap polyposis occurs as multiple sessile polyps, however, it can present as a single solitary sessile polyp as seen in our case in the sigmoid region. It usually occurs late in life but incidence had been reported to occur as early as twelve years and up to seventy-six years of age.^4^ Recently, other factors have been proposed as potential causes, including dysbiosis of the gut microbiota, persistent mucosal irritation brought on by straining during faeces, mucosal prolapse related to colonic dysmotility, and prior pelvic surgery.^5^

A past study described an infectious aetiology based in part upon symptomatic and endoscopic improvement after treatment of Helicobacter pylori with antibiotics.^6^ However, *Helicobacter pylori* has not been isolated within the inflammatory colonic polyps. Postulated mechanisms by which gastric *Helicobacter pylori* causes extra-gastric cap polyposis include molecular mimicry and the release of inflammatory mediators.^6^ But, we didn't perform the *Helicobacter pylori* test. A similar study described a patient in whom a cap polyp was noted to progress along the surgical anastomotic line after a laparoscopic sigmoid colectomy was performed, signifies that local inflammation plays a role in the development of cap polyposis. The effectiveness of infliximab in two case reports also supports the role of inflammation in the pathogenesis of cap polyposis. On reviewing the literature various age groups have different presentations. Most children with cap polyposis presented with common symptoms like rectal bleeding (100%), constipation (53%), diarrhoea (40%), and abdominal pain (40%). Whereas, the most common symptoms among adults are mucous discharge/diarrhoea (87%), bloody stool (33%), weight loss (10%), abdominal pain (10%), and tenesmus (10%).^7^

The natural course of cap polyposis is largely unknown. Reports range from spontaneous remission to recurrence after surgical resection, especially in patients with multiple polyps. However, surgical resection in multiple settings remains the diagnostic and therapeutic modality of treatment. Thus, repeat colonoscopy has been recommended to monitor for persistent disease and possible progression. The need and timing of colonoscopy should be individualized based on the adequacy of resection, the number of polyps, and the presence of symptoms.^4^ In several other cases-reports, the use of aminosalicylates, anti-inflammatory agents like topical and systemic steroids, antibiotics like metronidazole, and TNF-alpha inhibitors like infliximab had been described but their use is still conflicting.^8^ In children, however, conservative measures are preferred.

In adults, polypectomy should be performed to alleviate symptoms when possible. For medical management, testing for Helicobacter pylori is recommended because positive outcomes have been observed after eradication therapy. Surgical resection can be considered if the disease persists or recurs despite medical treatment.
